# Intensity-Based Nonoverlapping Area Registration Supporting “Drop-Outs” in Terms of Model-Based Radiostereometric Analysis

**DOI:** 10.1155/2018/8538125

**Published:** 2018-05-03

**Authors:** Ondrej Klima, Petr Novobilsky, Roman Madeja, David Barina, Adam Chromy, Michal Spanel, Pavel Zemcik

**Affiliations:** ^1^IT4Innovations Centre of Excellence, Brno University of Technology, Bozetechova 1/2, 612 66 Brno, Czech Republic; ^2^Radiology Institute, University Hospital in Ostrava, 17 Listopadu 1790, 708 52 Ostrava, Czech Republic; ^3^Trauma Center, University Hospital in Ostrava, 17 Listopadu 1790, 708 52 Ostrava, Czech Republic; ^4^Department of Control and Instrumentation, Brno University of Technology, Technicka 3082/12, 616 00 Brno, Czech Republic

## Abstract

A model-based radiostereometric analysis (MBRSA) is a method for precise measurement of prosthesis migration, which does not require marking the implant with tantalum beads. Instead, the prosthesis pose is typically recovered using a feature-based 2D-3D registration of its virtual model into a stereo pair of radiographs. In this study, we evaluate a novel intensity-based formulation of previously published nonoverlapping area (NOA) approach. The registration is capable of performing with both binary radiographic segmentations and nonsegmented X-ray images. In contrast with the feature-based version, it is capable of dealing with unreliable parts of prosthesis. As the straightforward formulation allows efficient acceleration using modern graphics adapters, it is possible to involve precise high-poly virtual models. Moreover, in case of binary segmentations, the nonoverlapping area is simply interpretable and useful for indicating the accuracy of the registration outcome. In silico and phantom evaluations were performed using a cementless Zweymüller femoral stem and its reverse engineered (RE) model. For initial pose estimates with difference from the ground-truth limited to ±4 mm and ±4°, respectively, the mean absolute translational error was not higher than 0.042 ± 0.035 mm. The error in rotation around the proximodistal axis was 0.181 ± 0.265°, and the error for the remaining axes was not higher than 0.035 ± 0.037°.

## 1. Introduction

Radiostereometric analysis (RSA), introduced by Selvik [1, 2], is an established method for an accurate measurement of prosthesis mechanical stability, indicated in particular in cases of total joint arthroplasty. The analysis is used for measuring micromotion between the prosthesis and the surrounding bone. Due to its high precision, it allows to reveal a potential failure of the implant fixation at early stages, when the prosthesis migration is not recognizable in plain radiographs, nor clinical symptoms occur [3]. The conventional radiostereometric analysis depends on two sets of tantalum beads. The first set of markers is attached to the prosthesis, while the second set of beads is injected directly into a bone surrounding the implant. The position of each marker in three-dimensional space is obtained using a triangulation from a stereo pair of radiographs. Commonly, a patient undergoes several following-up radiographic examinations during a certain time period after the arthroplasty [4]. A potential failure of the prosthesis fixation is observed when the relative pose between the two sets of markers differs between the individual examinations.

However, the attachment of tantalum beads to the implant raises several potential issues. In radiographs, the prosthesis may occlude the attached beads, the marked implants are more expensive, and the strength of the prosthesis may be negatively affected. To overcome these difficulties, model-based radiostereometric analysis (MBRSA) has been proposed by Valstar et al. [5]. Instead of attaching the beads, the implant pose is recovered by 2D-3D registration of its virtual model into a stereo pair of radiographs. Several studies have revealed that the model-based radiostereometry reaches lower but acceptable accuracy in comparison with the conventional approach [6–8].

Registration methods used in radiostereometry are typically feature-based, exploiting edges detected in radiographs and a prosthesis outline obtained from the virtual model. Valstar et al. [5] proposed an approach based on nonoverlapping area (NOA) minimization, which required a complete outline of the prosthesis to be obtained from the radiographs. The major drawback of the method was an inability to handle unreliable parts of the detected outline, as there were significantly large dimensional differences between the actual prosthesis and its CAD model involved in the phantom evaluation. A following-up study, proposed by Kaptein et al. [9], enhanced the accuracy by using reversed engineering (RE) models of prosthesis instead of CAD models provided by a manufacturer and by improving the registration method to handle the unreliable parts of detected contours, referred to as “drop-outs,” which may be caused by metallic objects that are not included in the virtual model, such as bone screws. The registration was based on minimization of contour difference, which can be in contrast with the original nonoverlapping area evaluated locally, and the unreliable parts of the contour, selected by the user, may be simply omitted from the registration. The minimization of contour difference was in broader principle adopted by many subsequent studies [6, 7, 10, 11].

In this study, we propose an intensity-based radiostereometric method, reviving the idea of nonoverlapping area. In contrast with [5], the proposed registration allows to evaluate the nonoverlapping area locally. Consequently, the contribution of this revisited method is the ability to handle the drop-outs and unreliable parts of the prosthesis captured in radiographs. As the contour detection and a feature matching are not required by the intensity-based registration, the computation is much more simple in comparison with the previously published approaches. Therefore, the method is straightforward for efficient acceleration using graphics adapters. The study presents in silico and phantom evaluations of the proposed approach.

## 2. Materials

**Figure 1:**
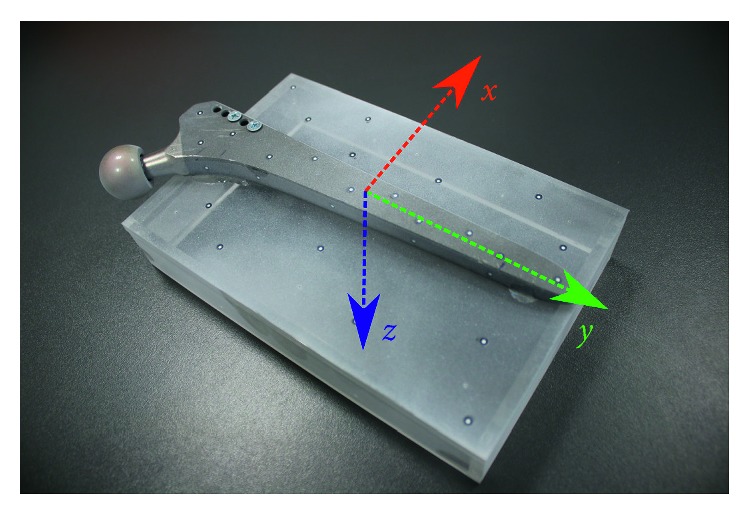
The Zweymüller stem attached to the Plexiglas phantom. The green arrow shows a proximodistal axis of the implant.

**Figure 2:**
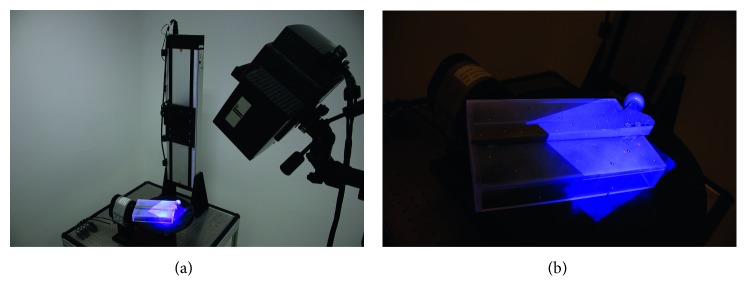
Scanning of the phantom assembly.

The study was performed involving Zweymüller SLR-PLUS Cementless Revision Stem produced by Smith & Nephew, Inc. The femoral stem was attached to a phantom containing 10 tantalum beads of 1 mm diameter provided by X-medics Scandinavia. The phantom was a box with dimensions 200 × 130 × 30 mm, created from extruded Plexiglas® of 6 mm thickness by the manufacturer Koplast s.r.o.. A complete assembly is shown in Figure 1. A polygonal model of the implant was generated using ATOS Triple Scan II system and ATOS Professional v8 SR1 software. The final model used for both in silico and phantom evaluations was formed by 236,053 vertices and 470,337 polygons. A mutual pose of the implant and the phantom box was determined by additional scanning of the assembly, as shown in Figure 2. Final positions of the tantalum markers with respect to the prosthesis were calculated, as their locations inside the phantom were defined by the CAD model, used for manufacturing the box.

**Figure 3:**
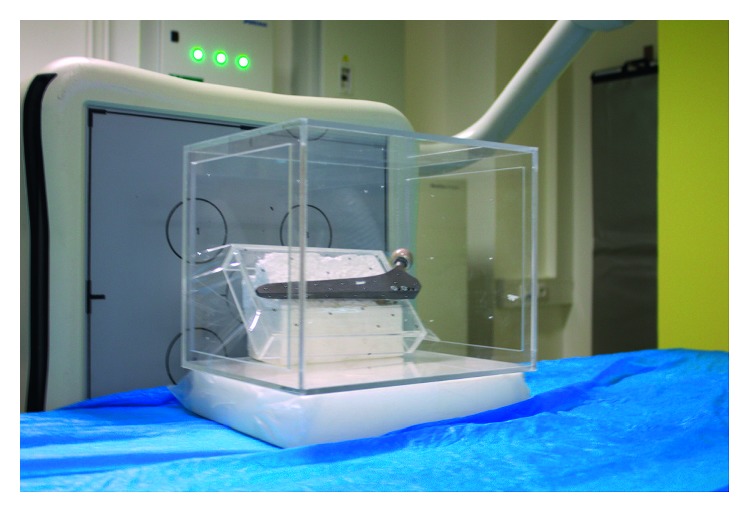
The phantom with attached prosthesis inside the calibration box, placed into the uniplanar imaging system. The phantom was firmly fixed to the calibration box.

The Carestream Directview DR 9500 System was exploited for sequential capturing of digital radiographs (DR). The phantom assembly was inserted into a biplanar calibration cage filled with 36 tantalum beads. We used direct linear transformation (DLT) [12] for the radiograph calibration, as proposed by Choo and Oxland [13], instead of the traditional fiducial and control planes (FCP) approach [2]. The assembly was rotated approximately 45° around the prosthesis proximodistal axis to prevent occlusions of the phantom markers by the implant. The complete experimental setup is shown in Figure 3.

**Figure 4:**
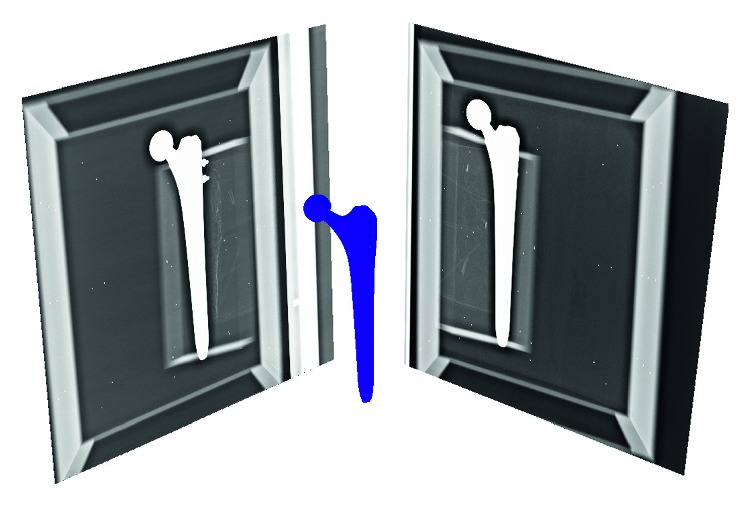
A stereo pair of enhanced radiographs with the RE model of the replacement. The model is in the ground-truth pose, determined using tantalum beads inside the phantom.

For the phantom study, 8 radiographs were captured from each anterior-posterior and lateral views. The pose of the calibration box within the imaging system was varied among the individual acquirements. The radiographs were enhanced using an intensity curve adjustment and histogram equalization. Upon the radiographs, a set of 64 stereo pairs was constructed, and an example stereo pair of radiographs is shown in Figure 4. Randomly chosen 32 pairs were exploited for a precise refinement of the mutual pose between the phantom and the implant, and the remaining half was used for the evaluation.

## 3. Methods

The proposed registration is suitable for usage with both binary segmentations and enhanced nonsegmented radiographs.

### 3.1. Binary Images

**Figure 5:**
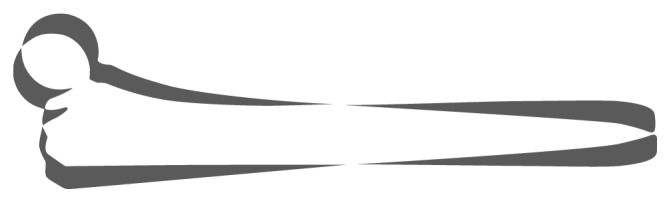
Nonoverlapping area between the actual and reprojected prosthesis segmentation. The error is set to *R*_err_=(4.59,   − 3.68,   − 2.38)° and *T*_err_=(−1.31,  1.07,   − 1.03) mm. The size of the nonoverlapping area is 27.5%.

As the metallic implants are highly radiopaque, the segmentation is performed by thresholding the enhanced radiographs; pixels representing the prosthesis are set to 1. A coarse initial estimate of the prosthesis pose must be provided by the user. During the registration, binary digitally reconstructed radiographs (DRR) are rendered from the prosthesis model. Following [5], the nonoverlapping area is defined as the area that the segmentations of real and calculated radiographs do not have in common. The size of the area is equal to the count of different pixels between the real and virtual segmentations. Since the segmentations contain only binary values, the count is computed by summing squares of the pixel differences (PD):

(1) $$\begin{array}{c} \begin{array}{l} \mathrm{PD}\left(p,\;x,\;y\right)=\mathrm{DR}\left(x,\;y\right)-\mathrm{DRR}\left(p,\;x,\;y\right),\\ \mathrm{NOA}\left(p\right)=\sum_{x,y}{\left\|\mathrm{PD}\left(p,\;x,\;y\right)\right\|}^{2}, \end{array} \end{array}$$

where *𝓅*=(*R*,  *T*) is a vector formed by a rotation and translation of the prosthesis model in the space of stereo radiographs. To eliminate different radiograph resolutions or perspective scaling, it is convenient to express the nonoverlapping area size in a relative form as NOA(*𝓅*)/(NOA(*𝓅*)+*C*(*𝓅*)), where *C*(*𝓅*) is a count of overlapping pixels. The metric is schematically depicted in Figure 5. The minimization of the nonoverlapping area for anterior-posterior (AP) and lateral (LAT) views is formulated as nonlinear least-squares (NLS) problem:

(2) $$\begin{array}{c} \left(p^{*}\right)=\underset{p}{\arg\;\;\min}\left[\mathrm{NO}A_{\mathrm{AP},\mathrm{LAT}}\left(p\right)\right]. \end{array}$$

### 3.2. Nonsegmented Radiographs

Due to the significant radiopacity, it is assumed that the metallic prostheses are objects with the highest contrast in radiographs, exceeding the brightness of the surrounding bone, soft tissues, or eventual cement layer, which makes the segmentation rather a straightforward task. On the other hand, a precise segmentation may demand some additional user interaction, and consequently, to decrease the amount of required user efforts, it is convenient to perform the registration using directly the nonsegmented radiographs. In case of the proposed intensity-based registration, radiographs are preprocessed using the histogram equalization. After the preprocessing, pixels representing the prosthesis reach approximately the maximum value of the image intensity range. The digitally reconstructed radiographs contain only two intensity levels. Following the radiopacity assumption, the virtual model is rendered with the highest contrast, while the background pixels are set to the lowest intensity. As the intensity-based nonoverlapping area registration is formulated as a least-squares problem, it is clearly suitable for usage with gray-scale images. With respect to the high prosthesis radiopacity and the least-squares formulation of the registration, the equalized radiographs may be considered as a probabilistic approximation of the prosthesis segmentation. However, in this case, the sum of squared differences does not correspond to the exact size of the nonoverlapping area, in contrast with the registration involving only the binary segmentations.

### 3.3. Handling Drop-Outs

**Figure 6:**
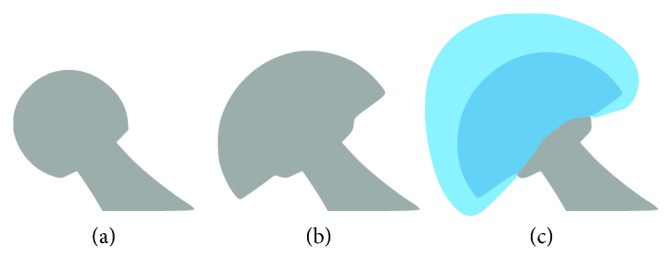
A femoral stem with attached ball head (a), ball head occluded by the acetabular prosthesis (b), and blue-colored drop-out area roughly selected by a user (c).

Drop-outs are especially related with metallic objects that are not a part of the prosthesis virtual model, but which are present in radiographs and occlude certain parts of the implant. In case of hip prosthesis, the ball head attached to the femoral stem may be occluded by a metallic acetabular implant. In this case, a user must roughly select the area, where a boundary of the prosthesis, corresponding to the virtual model, is unclear. The situation is schematically illustrated in Figure 6. Consequently, the drop-out areas are discarded from both input X-ray images and digitally reconstructed radiographs; hence, they do not affect the registration accuracy. The drop-outs are supported by both segmentation-based and intensity-based registrations. However, they were not supported by the original contour-based approach [5], as it required a complete and precise outline of the prosthesis to be extracted from the input radiographs.

### 3.4. Optimization Scheme

During the registration, 6 degrees of freedom (DOF) of the model pose are optimized using Levenberg–Marquardt numerical solver [14]. As the Levenberg–Marquardt optimization is gradient based, an evaluation of Jacobian matrix *J*_F_ is required during each iteration [15]. The matrix contains partial derivatives of pixel differences with respect to the pose parameters:

(3) $$\begin{array}{c} J_{F}=\left.\left(\begin{array}{cc} \frac{\partial \mathrm{PD}\left(p,\;1,\;1\right)}{\partial \left[r_{x},\;r_{y},\;r_{z}\right]} & \frac{\partial \mathrm{PD}\left(p,\;1,\;1\right)}{\partial \left[t_{x},\;t_{y},\;t_{z}\right]}\\ \vdots & \vdots \\ \frac{\partial \mathrm{PD}\left(p,\;x,\;y\right)}{\partial \left[r_{x},\;r_{y},\;r_{z}\right]} & \frac{\partial \mathrm{PD}\left(p,\;x,\;y\right)}{\partial \left[t_{x},\;t_{y},\;t_{z}\right]} \end{array}\right)\right\}w_{\mathrm{AP}}h_{\mathrm{AP}}+w_{\mathrm{LAT}}h_{\mathrm{LAT}}. \end{array}$$

The number of rows in *J*_F_ matrix is given by the total count of pixels in both anterior-posterior and lateral images, and the number of columns is equal to the count of degrees of freedom. As it is not possible to evaluate the Jacobian matrix using a closed-form solution, we use a central difference approximation:

(4) $$\begin{array}{c} \frac{\partial }{\partial p}\mathrm{PD}\left(p,\;x,\;y\right)\approx \frac{1}{2\varepsilon }\mathrm{DRR}\left(p+\varepsilon ,\;x,\;y\right)-\frac{1}{2\varepsilon }\mathrm{DRR}\left(p-\varepsilon ,\;x,\;y\right), \end{array}$$

where *p* ∈ *𝓅* is a certain pose parameter and *ε* is a difference spacing. To increase both capture range and accuracy at the same time, the registration is divided into five subsequent optimizations where the coarse-to-fine strategy is applied on the different spacing *ε*. Stages with *ε* equal to 1*e*^1^, 1*e*^0^, 1*e*^−1^, 1*e*^−2^, and  1*e*^−3^ millimeters or degrees, respectively, were used in the study. To speed up the registration and lower the memory requirements, only regions of interest were cropped from radiographs to form the pixel differences vector, based on bounding boxes of the implant segmentations. To prevent an undesirable cropping of the nonoverlapping areas, the bounding boxes were enlarged by certain margins. Due to pixel-wise formulation of the registration, places containing drop-outs, selected by the user, were simply discarded from the registration.

## 4. Results

### 4.1. In Silico Evaluation

**Figure 7:**
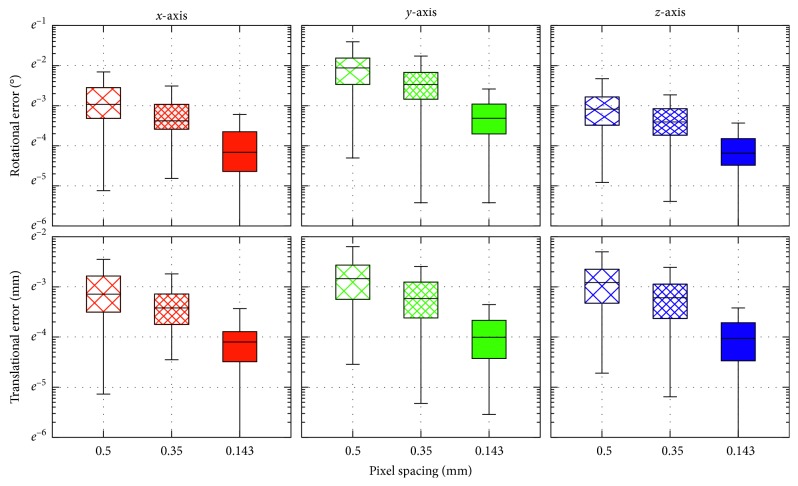
Distributions of absolute rotational and translational errors in dependence on the radiographs pixel spacing. The error distributions are shown in logarithmic scale.

As eventual segmentation errors may negatively affect the 2D-3D registration [16], the aim of the in silico evaluation was to investigate the intensity-based approach accuracy itself, without external influences. Three data sets containing one hundred virtual stereo radiographs of the implant, differing in resolution, were created with pixel spacings set to 0.5, 0.35, and 0.143 mm. The initial poses were generated randomly with uniform distribution, and the maximal translational and rotational errors were limited to ±5 mm and ±5°, respectively.  Table 1 shows mean values and standard deviations of absolute pose errors together with corresponding nonoverlapping area size, number of iterations, and processing time. A relation between accuracy and pixel spacing is shown using box plots in Figure 7. The accuracy obviously increases with the radiograph resolution, as the registration is able to perform more iterations. However, the rising count of iterations together with increasing length of the pixel differences (PD) vector yields into a trade-off between the registration accuracy and the processing time.

### 4.2. Phantom Evaluation

The model-based radiostereometric analysis monitors possible changes in relative pose between the bone and the joint replacement among certain time periods. The prosthesis pose is recovered by the registration of its virtual model into a stereo pair of radiographs, while the pose of the bone is obtained using a tantalum beads placed inside the bone. The tantalum beads are inserted using commercially available injectors, provided for instance by Tilly Medical Products AB or RSA Biomedical Suppliers. A three-dimensional pose of the bone markers is easily obtained from the stereo radiographs by triangulation. In consequence, the prosthesis migration is measured with respect to the set of markers injected into the bone. During the phantom study, for the accuracy evaluation purposes, the ground-truth pose of the implant within the space of stereo radiographs was determined using ten tantalum markers inside the phantom Plexiglas box, as the relative pose between the phantom and the attached prosthesis was known.

**Figure 8:**
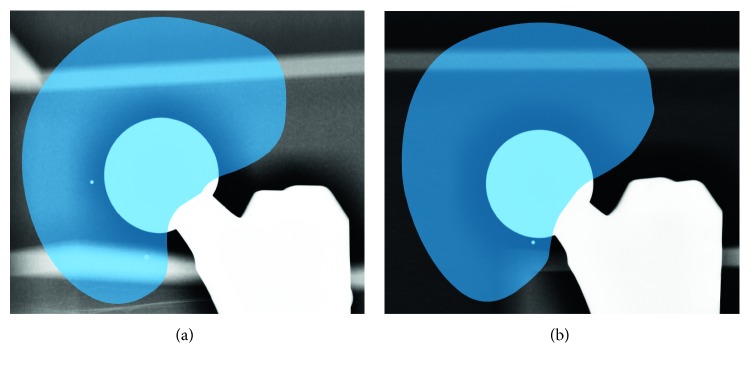
A sample stereo pair of radiographic images with roughly selected drop-out areas, chosen from the drop-outs evaluation data set. The selected areas, highlighted by blue overlay, are present in the places, where the femoral prosthesis with the attached ball head may be occluded by a metallic acetabular implant, as schematically shown in Figure 6.

**Figure 9:**
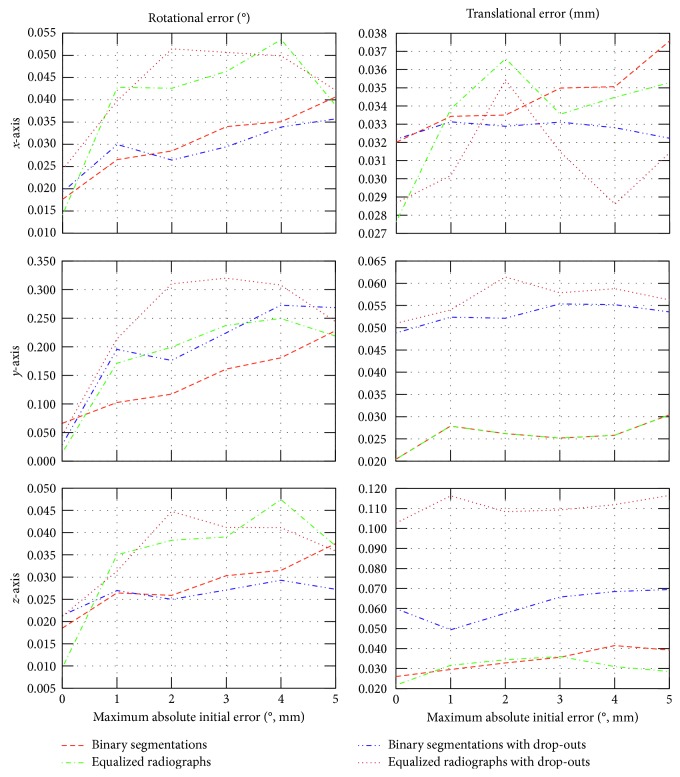
Dependence of the registration accuracy on the initial pose estimation error.

The registration was evaluated for both binary and nonsegmented radiographic images, with and without user selected drop-outs. A sample stereo pair containing drop-outs, chosen from the evaluation data set, is shown in Figure 8. The results of accuracy evaluations with and without drop-outs are shown in Tables 1 and 2, respectively, and their comparison is visualized in Figure 9. To investigate a relation between the capture range and the registration accuracy, the evaluations were performed for different limitations of maximal errors in initial poses, revealing a gentle decrease of accuracy and higher number of iterations with rising initial pose error.

The results show that the estimation of proximodistal rotation reaches the lowest accuracy in comparison with other pose parameters. The accuracy of the rotation around the *y*-axis would be increased by involving a third radiographic image taken in proximodistal projection, allowing the registration to minimize a nonoverlapping area even in the *xz* plane. However, in a real clinical environment, it is not possible to capture a radiographic image from such projection.

Generally, the recovery of the prosthesis pose using its virtual model is possible due to sufficient asymmetry of the implant, leading to unique projections of the model [5, 9]. Therefore, dropping the ball head out from the radiographs, a significantly asymmetric part of the prosthesis, which may be on the other hand in real situation occluded by a metallic acetabular implant, has rather slight but still recognizable influence on the registration accuracy. As the ball head is the most proximal and the most medial part of the model, a slight decrease of the accuracy can be seen mainly in the translation along the proximodistal and *z*-axis. The method also performs for binary segmentations with slightly higher accuracy than for enhanced radiographs.

The registration pipeline was implemented using Qt Toolkit 5.8.0 and compiled with MSVC 2013 64-bit. To speedup the registration, the rendering part of the pipeline was accelerated using OpenGL 4.3. The evaluations were performed using a Microsoft Windows 8.1 64-bit desktop machine equipped with an Intel Core i5-6500 CPU processor, an NVidia 980 GTX Ti 6GB graphics adapter, and a 24 GB DDR4 SDRAM memory module.

## 5. Discussion and Conclusion

In contrast with 2D-3D registration methods exploiting contour difference minimization, the nonoverlapping area does not require feature matching between detected and virtual contours, which is a computationally demanding and error-prone task. Considering theoretical aspects, determining matches between the contours is in principle an ill-posed problem. Strictly speaking, there are no actual correspondences between the detected and calculated contours until the ground-truth pose of the model is recovered. In other poses, the virtual contour captures different places of the prosthesis than the edges detected in radiographs. We therefore suggest that the nonoverlapping area has a stronger theoretical basis than the contour difference registration.

A computation of the intensity-based nonoverlapping area is more straightforward in comparison with the original feature-based formulation. In the feature-based case, the area was evaluated using nontrivial procedure based on horizontal directions of both detected and virtual contours [5]. On the contrary, the intensity-based variant exploits plain pixel differences between radiographs and virtual segmentations obtained from the prosthesis model.

As the OpenGL acceleration was focused only on the part of the pipeline, data transfers between a graphical and operational memory were a cause of a performance bottleneck. There is an opportunity for further significant acceleration by implementing the rest of the registration pipeline using the OpenGL compute shader programs, eliminating the memory transfers and exploiting parallelization of the Levenberg–Marquardt algorithm. We believe the shift from the feature to intensity-based variant is possible due to rapid progress of hardware performance, as the intensity-based registration feasibility depends on usage of modern hardware resources.

Due to efficient graphics hardware and intensity-based formulation, it is possible to involve complete high-poly RE models without decimating the mesh, in contrast with studies presented by Kaptein et al. and Seehaus et al. [9, 17]. The registration accuracy is comparable with previously published feature-based approaches, according to the summary presented by Syu et al. [11]. However, the comparison is rather tentative, as the accuracy depends on the shape of involved implants [6] and on the type of imaging system. An important contribution of the intensity-based revision is the ability to handle the drop-outs, which are useful for dealing with components that are not a fixed part of the prosthesis model. We also suggest that the relative size of the nonoverlapping area is a simply interpretable metric, useful for indicating the resulting accuracy of the registration.

## Figures and Tables

**Table 1:** Results of the accuracy and performance evaluations.

Maximum initial error (mm, °)	Spacing (mm)	Rotational error (mean ± SD)			Translational error (mean ± SD)			Iterations	Time (s)	NOA (%)
		*x* (°)	*y* (°)	*z* (°)	*x* (mm)	*y* (mm)	*z* (mm)			
*In silico evaluation*										
±5	0.5	0.006 ± 0.041	0.040 ± 0.170	0.006 ± 0.041	0.011 ± 0.010	0.022 ± 0.210	0.010 ± 0.081	24.6	5.2	0.197
±5	0.35	0.001 ± 0.002	0.005 ± 0.006	0.001 ± 0.008	0.001 ± 0.007	0.001 ± 0.001	0.001 ± 0.001	26.7	9.2	0.004
±5	0.143	0.000 ± 0.000	0.001 ± 0.001	0.000 ± 0.000	0.000 ± 0.000	0.000 ± 0.000	0.000 ± 0.000	31.8	44.4	0.000
*Phantom evaluation using binary segmentations*										
0	0.143	0.018 ± 0.015	0.066 ± 0.056	0.019 ± 0.013	0.032 ± 0.019	0.020 ± 0.010	0.026 ± 0.020	25.3	—	1.079
±1	0.143	0.027 ± 0.021	0.103 ± 0.070	0.026 ± 0.016	0.033 ± 0.019	0.028 ± 0.011	0.030 ± 0.023	54.5	—	1.080
±2	0.143	0.029 ± 0.022	0.117 ± 0.079	0.026 ± 0.018	0.034 ± 0.019	0.026 ± 0.011	0.033 ± 0.021	62.7	—	1.081
±3	0.143	0.034 ± 0.034	0.161 ± 0.230	0.030 ± 0.028	0.035 ± 0.025	0.025 ± 0.011	0.036 ± 0.028	71.3	—	1.088
±4	0.143	0.035 ± 0.037	0.181 ± 0.265	0.032 ± 0.030	0.035 ± 0.026	0.026 ± 0.011	0.042 ± 0.035	85.5	—	1.093
±5	0.143	0.041 ± 0.051	0.228 ± 0.424	0.038 ± 0.047	0.038 ± 0.030	0.030 ± 0.026	0.039 ± 0.025	98.5	108.7	1.121
*Phantom evaluation using enhanced radiographs*										
0	0.143	0.014 ± 0.016	0.014 ± 0.011	0.010 ± 0.010	0.028 ± 0.020	0.013 ± 0.009	0.022 ± 0.017	16.8	—	—
±1	0.143	0.043 ± 0.048	0.171 ± 0.192	0.035 ± 0.037	0.034 ± 0.024	0.028 ± 0.010	0.032 ± 0.023	43.6	—	—
±2	0.143	0.043 ± 0.062	0.199 ± 0.284	0.038 ± 0.056	0.037 ± 0.033	0.028 ± 0.012	0.034 ± 0.022	84.3	—	—
±3	0.143	0.046 ± 0.067	0.237 ± 0.376	0.039 ± 0.063	0.034 ± 0.032	0.031 ± 0.018	0.036 ± 0.026	101.4	—	—
±4	0.143	0.054 ± 0.078	0.250 ± 0.436	0.047 ± 0.076	0.035 ± 0.035	0.033 ± 0.024	0.031 ± 0.026	113.5	—	—
±5	0.143	0.039 ± 0.069	0.219 ± 0.403	0.037 ± 0.068	0.035 ± 0.036	0.031 ± 0.023	0.029 ± 0.020	120.8	188.0	—

**Table 2:** Accuracy and performance evaluation of phantom data containing drop-outs.

Maximum initial error (mm, °)	Spacing (mm)	Rotational error (mean ± SD)			Translational error (mean ± SD)			Iterations	Time (s)	NOA (%)
		*x* (°)	*y* (°)	*z* (°)	*x* (mm)	*y* (mm)	*z* (mm)			
*Evaluation using binary segmentations*										
0	0.143	0.019 ± 0.015	0.029 ± 0.031	0.021 ± 0.014	0.032 ± 0.018	0.049 ± 0.015	0.060 ± 0.033	22.7	—	1.090
±1	0.143	0.030 ± 0.021	0.195 ± 0.155	0.027 ± 0.016	0.033 ± 0.017	0.052 ± 0.018	0.049 ± 0.034	47.0	—	1.108
±2	0.143	0.026 ± 0.024	0.176 ± 0.154	0.025 ± 0.018	0.033 ± 0.018	0.052 ± 0.014	0.058 ± 0.039	60.1	—	1.104
±3	0.143	0.029 ± 0.024	0.224 ± 0.174	0.027 ± 0.017	0.033 ± 0.019	0.055 ± 0.020	0.066 ± 0.058	68.2	—	1.120
±4	0.143	0.034 ± 0.029	0.273 ± 0.322	0.029 ± 0.022	0.033 ± 0.021	0.055 ± 0.021	0.069 ± 0.058	79.5	—	1.150
±5	0.143	0.036 ± 0.027	0.268 ± 0.260	0.027 ± 0.018	0.032 ± 0.018	0.054 ± 0.024	0.070 ± 0.049	93.9	113.0	1.174
*Evaluation using enhanced radiographs*										
0	0.143	0.024 ± 0.022	0.045 ± 0.035	0.021 ± 0.021	0.029 ± 0.018	0.051 ± 0.017	0.103 ± 0.052	17.4	—	—
±1	0.143	0.040 ± 0.030	0.214 ± 0.201	0.031 ± 0.024	0.030 ± 0.019	0.054 ± 0.022	0.116 ± 0.064	41.5	—	—
±2	0.143	0.051 ± 0.047	0.310 ± 0.402	0.045 ± 0.046	0.035 ± 0.025	0.061 ± 0.019	0.108 ± 0.059	73.7	—	—
±3	0.143	0.051 ± 0.047	0.320 ± 0.389	0.041 ± 0.038	0.031 ± 0.024	0.058 ± 0.029	0.109 ± 0.053	90.0	—	—
±4	0.143	0.050 ± 0.049	0.308 ± 0.404	0.041 ± 0.036	0.029 ± 0.021	0.059 ± 0.028	0.112 ± 0.054	108.0	—	—
±5	0.143	0.042 ± 0.041	0.244 ± 0.327	0.036 ± 0.031	0.031 ± 0.021	0.056 ± 0.025	0.117 ± 0.051	115.0	188.8	—
